# Designing Potential Donor Materials Based on DRCN5T with Halogen Substitutions: A DFT/TDDFT Study

**DOI:** 10.3390/ijms222413498

**Published:** 2021-12-16

**Authors:** Yunjie Xiang, Jie Zhang, Shaohui Zheng

**Affiliations:** 1 School of Materials and Energy, Southwest University, Chongqing 400715, China; zjzj123123@swu.edu.cn; 2 Chongqing Key Laboratory for Advanced Materials and Technologies of Clean Energies, Southwest University, Chongqing 400715, China

**Keywords:** DFT, small organic solar cell, oligothiophenes, halogen substitutions, photovoltaic properties

## Abstract

Experimental researchers have found that the organic solar cell (OSC) based on DRCN5T (an oligothiophene) possesses excellent power conversion efficiency (PCE) of 10.1%. However, to date, there have been few studies about halogenation of DRCN5T, and its effects on photovoltaic properties of halogenated DRCN5T are still not clear. In the present work, we first perform benchmark calculations and effectively reproduce experimental results. Then, eight halogenated DRCN5T molecules are designed and investigated theoretically by using density functional theory (DFT) and time-dependent DFT. The dipole moments, frontier molecular orbital energies, absorption spectra, exciton binding energy (E_b_), singlet–triplet energy gap (ΔE_ST_), and electrostatic potential (ESP) of these molecules, and the estimated open circuit voltages (V_OC_s) of the OSCs with PC_71_BM as acceptor are presented. We find that (1) generally, halogen substitutions would increase V_OC_; (2) E_b_ rises with more fluorine substitutions, but for Cl and Br substitutions, E_b_ increases firstly and then drops; (3) ΔE_ST_ keeps increasing with more halogen substitutions; (4) except for Br substitutions, the averaged ESP arises along with more halogen substitutions; (5) the absorption strength of UV–Vis spectra of DRCN5T2F, DRCN5T4F, DRCN5T6F, and DRCN5T2Cl in the visible region is enhanced with respect to DRCN5T. Based on these results, overall, DRCN5T2Cl, DRCN5T4F, and DRCN5T6F may be promising donors.

## 1. Introduction

**Figure 1:**
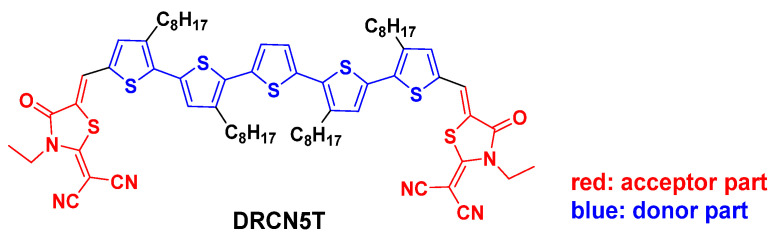
Molecular structure of DRCN5T.

Compared to polymer donors, small molecule donors possess several advantages such as easy purification, well-defined structures, and negligible batch-to-batch inconsistency. Oligothiophenes, as organic semiconductor materials, have been widely used in organic photovoltaics (OPVs) because they have excellent charge transport properties, tunable optical/electrochemical properties, high polarizability and high stability, relatively simple synthesis, and easy access [1,2,3,4,5]. Researchers have fabricated different OSCs based on oligothiophenes, and the PCEs of these OSCs are in the range of 1.49–10.1% [4,5,6,7]. Specifically, Chen and his co-workers have synthesized DRCN7T, a linear A-D-A (A: acceptor; D: donor) type of molecule with septithiophenes as a skeleton and 2-(1,1-dicyanomethylene) rhodanine as two end groups, and the organic solar cell (OSC) based on this synthesis has power conversion efficiency (PCE) of 9.3% [6]; later they reported a series of A-D-A molecules with different lengths of skeleton and the same end groups, namely DRCNnT (*n* = 4~9), and they found that the conjugation length and spatial symmetry greatly affect molecular packing, phase separation, molecular orbital energy levels, and charge carrier mobility [7]. The most striking finding is that the OSC with DRCN5T (Figure 1) as a donor and PC_71_BM as a acceptor achieves the PCE of 10.1%.

Halogenation of organic molecules has been proved to be capable of improving opto-electronic performances: firstly it can increase the open circuit voltage (V_OC_), which should be attributed to the deeper highest occupied molecular orbital (HOMO) energy level [8,9,10,11,12,13]; secondly, halogenations may improve the morphology of the active layer [14]. Peng’s group [15] tuned the energetic structure of two polymers (PvBDT-F and PvBDT-Cl) via halogenation, and suitable absorption ranges and frontier molecular orbital energy levels were obtained when pairing with high performance NFA molecule Y6-T. The OSC with chlorinated PvBDT-Cl showed higher photovoltaic performances (PCE = 8.23%) than that with fluorinated PvBDT-F. Lu’s group [16] chlorinated the original BTR donor molecule, which possessed strong intermolecular interaction, low-lying molecular energy levels, and a strong stacking tendency of edge-on orientation. Because of the different molecular packing orientations and different liquid crystalline properties between donor and acceptor, an appropriate morphology was achieved in the BTR-Cl:Y6 system, leading to a high PCE of 13.6% for all-small-molecule OSCs. Frequently, fluorine (F) and chlorine (Cl) atoms are introduced in donors to adjust the electronic structure and intermolecular interactions to obtain more efficient donors. So far, there are few studies about halogenation of DRCNnT. Beaujuge’s group [17] reported a bulk-heterojunction solar cell with V_OC_ over 1.10 V with di-fluorinated DRCN5T as a donor and PC_71_BM as an acceptor. Lu’s group [18] studied the isomeric effect of fluorine substitution on the performance of solar cells based on di-fluorinated DRCN5T (donor):IDIC4F (acceptor). However, until now, studies about fluorination of DRCN5T have lacked clarity and theoretical insights. Furthermore, there is no research about chlorine and bromine substitutions of DRCN5T. In brief, a systematic study of halogenation of DRCN5T is needed to consider the potential of DRCN5T.

**Figure 2:**
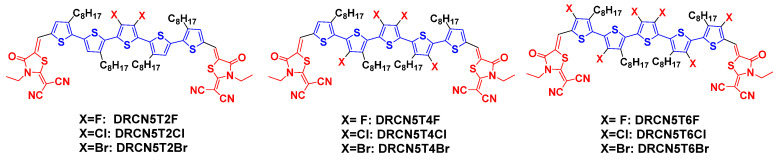
Molecular structures of DRCN5T2X, DRCN5T4X, and DRCN5T6X (X = F, Cl, and Br).

Therefore, in this work, we have systematically modelled new molecules DRCN5T4F, DRCN5T6F, DRCN5T2Cl, DRCN5T4Cl, DRCN5T6Cl, DRCN5T2Br, DRCN5T4Br, and DRCN5T6Br, as shown in Figure 2. These molecules are rationally and systematically designed with different numbers of halogen atoms. For instance, DRCN5T2Cl and DRCN5T2Br have exactly the same substituted positions as DRCN5T2F. Similarly, the halogen substitutions of DRCN5T4X and DRCN5T6X are highly possible and should be synthesized in experiments. By substitutions with halogen atoms, we have modified the chemical structure, charge distribution, and planarity of these molecules. We plan to investigate the effects on the photovoltaic performance of these molecules. We utilize density functional theory (DFT) and time-dependent DFT (TDDFT) to study how different halogen substitutions influence opto-electronical properties such as dipole moments, frontier molecular orbitals (FMOs), energy gap, UV–Vis spectra, and V_OC_s (with PC_71_BM as acceptor), exciton binding energy, ΔE_ST_, and ESP of these molecules. The theoretical studies about predicting the opto-electronic properties of newly designed small photovoltaic molecules have been shown to be quite reliable [19].

In particular, the dipole moment of a small molecule is important. With a proper dipole moment, relatively good charge separation and stacking morphology can be obtained to achieve high PCE [18]. Researchers have investigated the triplet behavior of DRCN5T [20], but they have not studied the effect of ΔE_ST_ (energy of S_1_ minus energy of T_1_) on DRCN5T and its halogenated derivatives to further understand the importance of triplet state. ΔE_ST_ is a crucial parameter for developing organic photovoltaic materials. Small ΔE_ST_ generally decreases the charge recombination in exciton dissociation and voltage loss. So here it is important to investigate the above properties.

The purposes of this work are twofold:

(1) To investigate the effects of different halogen substitutions and the extent of halogenation on the photovoltaic properties of DRCN5T.
(2) To find new promising donors (if possible) based on DRCN5T.

## 2. Results and Discussion

### 2.1. Benchmark Calculations

**Figure 3:**
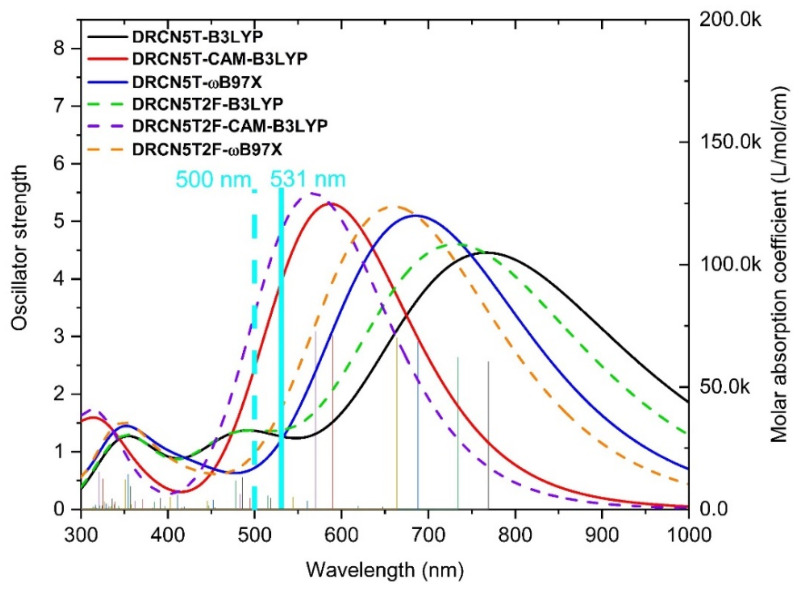
Simulated UV–Vis spectra of DRCN5T and DRCN5T2F with FWHM of 0.6667 eV based on different functionals with 6−31+G(d) basis set (black curve: B3LYP for DRCN5T; red: CAM-B3LYP for DRCN5T; blue: ωB97X for DRCN5T; green: B3LYP for DRCN5T2F; purple: CAM-B3LYP for DRCN5T2F; yellow: ωB97X for DRCN5T2F; line in light blue: experimental λ_max_ of DRCN5T; dashed line in light blue: experimental λ_max_ of DRCN5T2F) in chloroform.

We make use of CAM-B3LYP, tuned ωB97X, and B3LYP density functionals [21] with 6−31+G(d) to run TDDFT calculations of DRCN5T and DRCN5T2F molecules. Figure 3 shows the results of absorption spectra of DRCN5T and DRCN5T2F, based on the TDDFT outputs of CAM-B3LYP, B3LYP, and tuned ωB97X density functionals in chloroform (same as the experiment). The simulated absorption spectra of DRCN5T and DRCN5T2F with CAM-B3LYP functional are the closest to the experimental ones in the visible region. The wavelength of experimental absorption peaks of DRCN5T and DRCN5T2F is located at 531 nm and 500 nm, respectively [7,18]. As shown in Table S1, the wavelength of the absorption peaks of DRCN5T and DRCN5T2F obtained with CAM-B3LYP functional is located at 589 and 569 nm, respectively. However, for the results of ωB97X and B3LYP, the wavelengths of absorption peaks of both molecules are 688/663 nm and 766/731 nm, respectively. Obviously, CAM-B3LYP is superior to the other two density functionals. Regarding these data in Table 1, we conclude that the CAM-B3LYP is suitable for excited state calculations.

Based on the comparisons between experiments and the calculated data, we thus choose CAM-B3LYP as the most accurate functional to calculate DRCN5T, DRCN5T2F, and eight new designed halogenated derivatives.

### 2.2. Dipole Moments of the Halogenated Molecules

**Figure 4:**
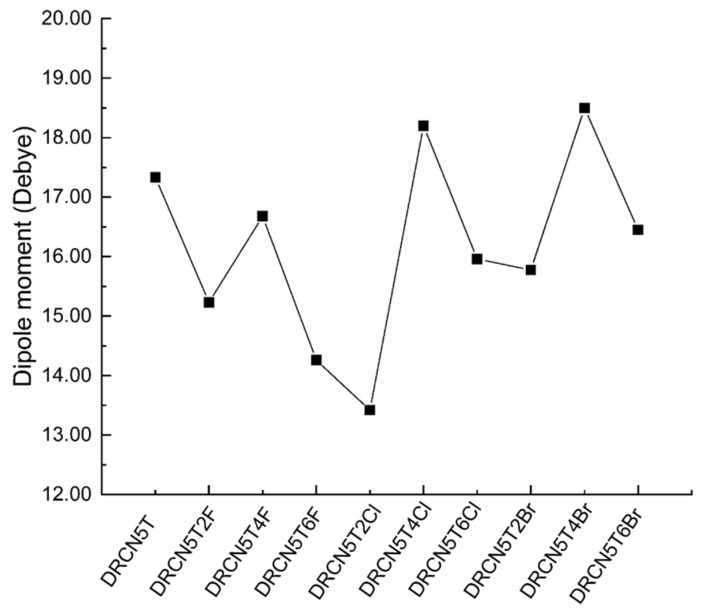
The calculated dipole moments of DRCN5T, DRCN5T2F, DRCN5T4F, DRCN5T6F, DRCN5T2Cl, DRCN5T4Cl, DRCN5T6Cl, DRCN5T2Br, DRCN5T4Br, and DRCN5T6Br gained with CAM-B3LYP/6−31+G(d) in film.

Dipole moments are related to the molecular packing and charge separation [22]. When the dipole moment increases, the charge separation is enhanced. However, if the dipole moment is too large, the molecules will aggregate unnecessarily, so the value of the dipole moment should be appropriate. As shown in Figure 4, DRCN5T, DRCN5T2F, DRCN5T4F, DRCN5T6F DRCN5T2Cl, DRCN5T4Cl, DRCN5T6Cl, DRCN5T2Br, DRCN5T4Br, and DRCN5T6Br possess the dipole moments of 17.33, 15.23, 16.68, 14.26, 13.42, 18.20, 15.96, 15.78, 18.50, and 16.45 Debye, respectively. DRCN5T has been proved to exhibit effective molecular packing between molecules [7]. However, it can be further improved. One good example is DRCN5T2F, synthesized by Lu and co-workers [18], which has a dipole moment of 15.23 Debye, slightly smaller than that of DRCN5T (17.33 Debye). Consequently, it does not aggregate as much as DRCN5T in experiments (Root-mean-square surface roughness is 3.15 nm for DRCN5T:IDIC-4F, only 2.43 nm for DRCN5T2F:IDIC-4F by AFM), enhancing the fill factor and short circuit current [18]. The PCE of the OSC with IDIC-4F as an acceptor and DRCN5T2F as donor was increased to 9.36% (the PCE of the OSC based on DRCN5T/IDIC-4F is 8.02%) [18]. As shown in Figure 4, DRCN5T2F, DRCN5T4F, DRCN5T6F DRCN5T2Cl, DRCN5T6Cl, DRCN5T2Br, and DRCN5T6Br have slightly smaller dipole moments (15.23, 16.68, 14.26, 13.42, 15.96, 15.78, and 16.45 Debye, respectively) than that of DRCN5T (17.33 Debye), which may benefit a higher PCE [23,24]. 

In Figure 4, DRCN5T2F (chemical structure is shown in Figure 2) has a smaller dipole moment than that of DRCN5T because two electron-withdrawing F atoms (two F at up positions on thiophene ring) shorten the distance between positive and negative charge centers of DRCN5T2F. From DRCN5T2F to DRCN5T4F to DRCN5T6F, the dipole moment first increases and then drops because four electron-withdrawing F atoms (two at up and two at down positions on thiophene rings) make the distance between the opposite charge centers longer, and six electron-withdrawing F atoms (four at up and two at down positions on thiophene rings) shorten the distance again. For Cl- and Br-substituted molecules, their dipole moments have the same trends. DRCN5T2Cl has the smaller dipole moment than that of DRCN5T2F owing to the stronger electron-withdrawing effects of 2Cl relative to 2F, which makes the distance between opposite charge centers shorter. DRCN5T2Br possesses the biggest dipole moment among DRCN5T2F, DRCN5T2Cl, and DRCN5T2Br on account of the weakest electron-withdrawing effects of 2Br.

### 2.3. LUMO/HOMO Energy and Energy Gap of the Halogenated Molecules

**Figure 5:**
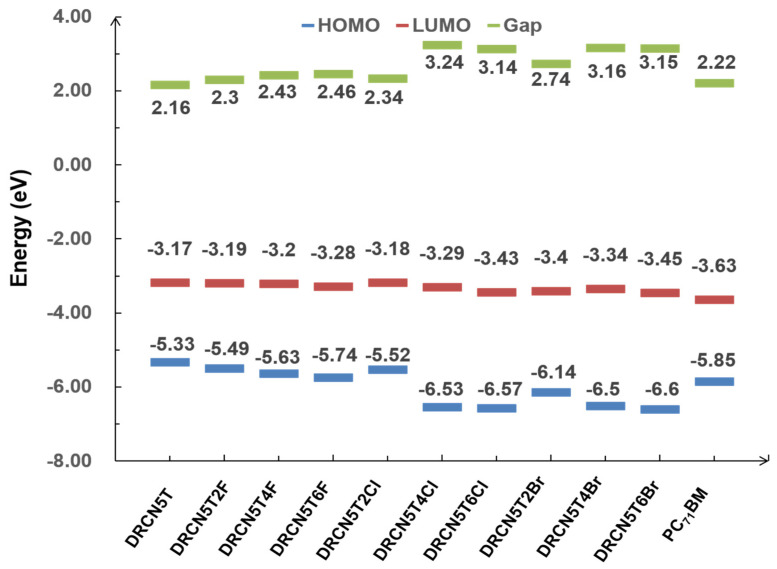
Calculated LUMO, HOMO and gap of DRCN5T, DRCN5T2F, DRCN5T4F, DRCN5T6F, DRCN5T2Cl, DRCN5T4Cl, DRCN5T6Cl, DRCN5T2Br, DRCN5T4Br, and DRCN5T6Br by using CAM-B3LYP/6−31+ G(d) in dichloromethane.

DRCN5T is substituted by different numbers of halogens to generate new molecules: DRCN5T4F, DRCN5T6F DRCN5T2Cl, DRCN5T4Cl, DRCN5T6Cl, DRCN5T2Br, DRCN5T4Br and DRCN5T6Br, respectively. Figure 2 shows the chemical structures of the eight new halogenated molecules. We utilize CAM-B3LYP density functional to calculate LUMO/HOMO energy and energy gap of the eight molecules. The results are displayed in Figure 5. The experimental HOMO/LUMO of DRCN5T and DRCN5T2F are −5.22 eV/−3.41 eV [7] and −5.34 eV/−3.59 eV, respectively [18]. Comparing the calculated data with the experimental values further confirms the accuracy of our chosen methods. The HOMO energy of the eight new molecules becomes lower after halogenation and the energy gaps become bigger than that of DRCN5T because of the electron-withdrawing ability of halogens. Additionally, the value of HOMO of DRCN5T4Cl, DRCN5T6Cl, DRCN5T2Br, DRCN5T4Br, and DRCN5T6Br drops a lot, which results in a significant increase in their V_OCS_. The energy gaps of DRCN5T4Cl, DRCN5T6Cl, DRCN5T2Br, DRCN5T4Br, and DRCN5T6Br are higher than the others (DRCN5T2F, DRCN5T4F, DRCN5T6F, DRCN5T2Cl) by at least 0.3 eV, which may be mainly attributed to their non-planarity [25] (see Figure S1). In Figure S1, the selected dihedral angles ψ1 and ψ2 of DRCN5T4Cl, DRCN5T6Cl, DRCN5T2Br, DRCN5T4Br, and DRCN5T6Br are between 77^o^ and 90^o^, indicating their strong non-planarity. While DRCN5T2F, DRCN5T4F, DRCN5T6F, and DRCN5T2Cl have planar structures. The radius of Cl and Br atoms are larger than that of F atoms, which causes the stronger non-planarity of DRCN5T after being substituted by them. 

### 2.4. V_OC_ of the OSC with Halogenated Molecules as Donors

Open circuit voltage (V_OC_) is one of important factors that influence the PCE of OSCs. We use the empirical formula reported by Scharber et al. to calculate V_OCS_ of OSCs based on these new molecules with PC_71_BM as acceptor [26]. The calculated LUMO/HOMO energy and energy gap of PC_71_BM by CAM-B3LYP is −3.01 eV, −5.78 eV, and 2.77 eV, respectively. Table 2 shows the calculated V_OC_ values of the OSCs with these new molecules as donors (utilizing Equation (10)). Due to the introduction of electron-withdrawing halogen atoms, the HOMO of the substituted molecule decreases, leading to the increase in V_OC_ (Equation (10)). The V_OC_ rises with the increasing number of the substituted halogen because of the stronger electron-withdrawing ability of halogens (DRCN5T2F < DRCN5T4F < DRCN5T6F, DRCN5T2Cl < DRCN5T4Cl < DRCN5T6Cl, DRCN5T2Br < DRCN5T4Br < DRCN5T6Br). Additionally, the V_OC_ becomes larger after chlorine and bromine substitutions. These results indicate that the type of the substituted halogen atoms has a great influence on V_OC_. 

### 2.5. Inner Reorganization Energy

**Figure 6:**
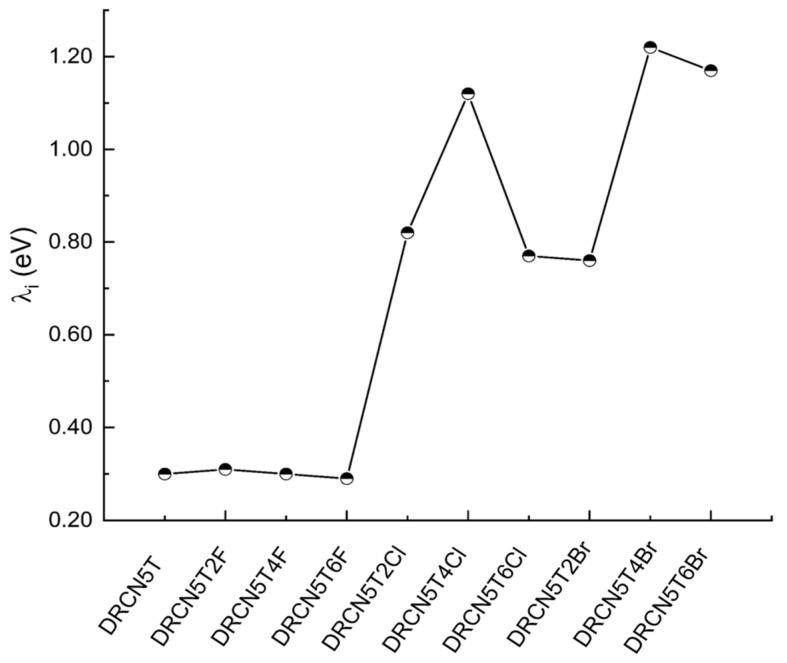
Calculated inner reorganization energies (eV) of DRCN5T, DRCN5T2F, DRCN5T4F, DRCN5T6F, DRCN5T2Cl, DRCN5T4Cl, DRCN5T6Cl, DRCN5T2Br, DRCN5T4Br, and DRCN5T6Br by using CAM-B3LYP/6−31+G(d) in film.

Inner reorganization energy (λ_i_) is the energy required for the change in geometric structure in the process of charge transport. The smaller the inner reorganization energy, the smaller the energy loss, and maybe the higher carrier mobility [27]. However, the carrier mobility is related to not only λ_i_, but also the external reorganization energy, the electronic coupling, and charge transfer distance [28]. The inner reorganization energy of DRCN5T, DRCN5T2F, DRCN5T4F, DRCN5T6F, DRCN5T2Cl, DRCN5T4Cl, DRCN5T6Cl, DRCN5T2Br, DRCN5T4Br, and DRCN5T6Br is calculated by using CAM-B3LYP/6−31+G(d) via Equation (12), as displayed in Figure 6. The inner reorganization energy of DRCN5T and these nine halogenated molecules is 0.30, 0.31, 0.30, 0.29, 0.82, 1.12, 0.77, 0.76, 1.22, 1.17 eV. DRCN5T2F, DRCN5T4F, and DRCN5T6F have almost the same level of inner reorganization energy as DRCN5T; DRCN5T6F has the smallest one (0.29 eV). The chlorine and bromine substituted DRCN5T derivatives have a much higher inner reorganization energy than that of DRCN5T and fluorine-substituted derivatives. 

### 2.6. Exciton Binding Energy of the Nine Halogenated Molecules

**Figure 7:**
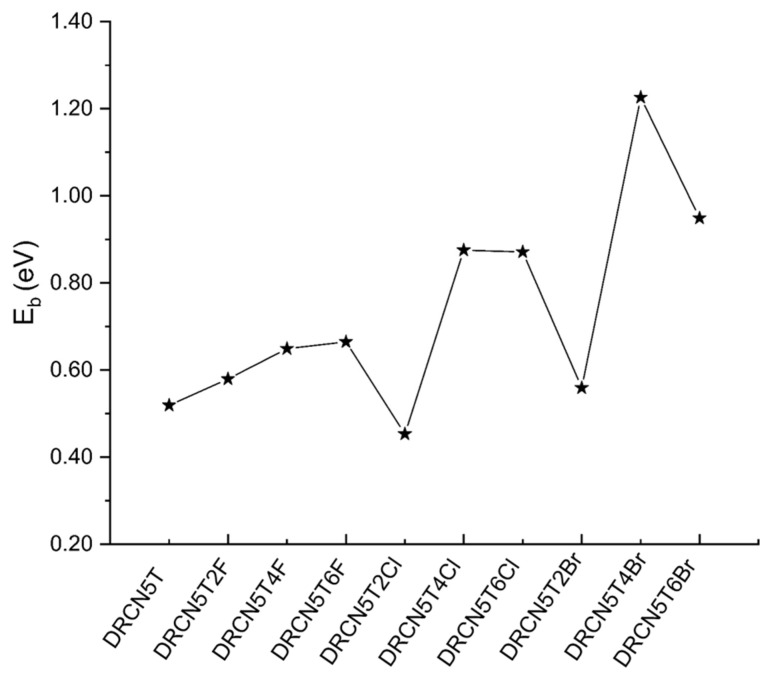
The calculated exciton binding energy of DRCN5T, DRCN5T2F, DRCN5T4F, DRCN5T6F, DRCN5T2Cl, DRCN5T4Cl, DRCN5T6Cl, DRCN5T2Br, DRCN5T4Br, and DRCN5T6Br with CAM-B3LYP/6−31+G(d) in film (ε=3.0).

Exciton binding energy is the energy used to separate excitons into hole and electron in organic solar cells. Small exciton binding energy should benefit J_SC_, charge separation, and PCE [29]. We present the calculated exciton binding energy of DRCN5T2F, DRCN5T4F, DRCN5T6F, DRCN5T2Cl, DRCN5T4Cl, DRCN5T6Cl, DRCN5T2Br, DRCN5T4Br, and DRCN5T6Br gained with CAM-B3LYP/6−31+G(d) in film. As shown in Figure 7 and Table S2, exciton binding energy is calculated by Equation (13). The exciton binding energy of DRCN5T and these nine molecules is 0.52, 0.58, 0.64, 0.67, 0.45, 0.87, 0.86, 0.56, 1.23 and 0.94 eV, respectively. The exciton binding energy of fluorine-substituted molecules increases with more fluorine substitutions. For Cl- and Br-substituted molecules, with the increase in the number of substitutions, the exciton binding energy first increases and then decreases. For DRCN5T2Cl, the fundamental gap (IP—EA) is 3.17 eV, and E_opt_ is 2.72 eV, so it obtains the lowest exciton binding energy of 0.45 eV; for DRCN5T4Br, the fundamental gap (IP—EA) increases to 4.45 eV, and its E_opt_ is 3.22 eV, therefore the exciton binding energy is the largest with the value of 1.23 eV (Table S2). DRCN5T2Cl possesses the smallest value of exciton binding energy, followed by DRCN5T, DRCN5T2Br, DRCN5T2F, DRCN5T4F, and then DRCN5T6F. The value of IP minus EA decreases, and it is closer to the optical gap, making the exciton binding energy of DRCN5T2Cl the smallest. Based on these results, DRCN5T2Cl molecule may be a potential donor material due to its small exciton binding energy.

### 2.7. Singlet–Triplet Energy Gap of the Nine Halogenated Molecules

**Figure 8:**
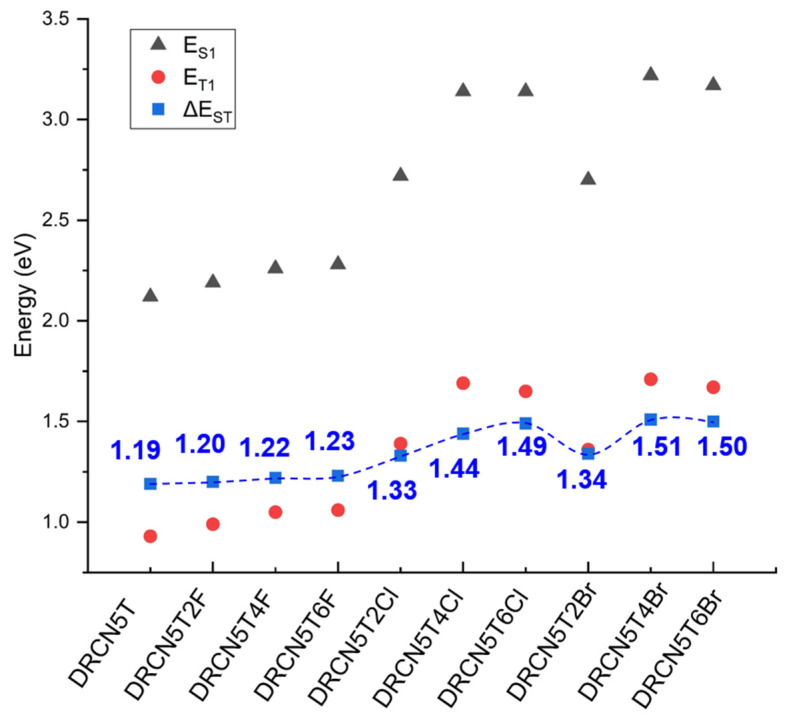
The lowest singlet (E_S1_), triplet excited energy (E_T1_) and singlet–triplet energy gap (ΔE_ST_) of DRCN5T, DRCN5T2F, DRCN5T4F, DRCN5T6F, DRCN5T2Cl, DRCN5T4Cl, DRCN5T6Cl, DRCN5T2Br, DRCN5T4Br, and DRCN5T6Br with CAM-B3LYP/6−31+G(d) in film (ε=3.0).

Singlet–triplet energy gap (ΔE_ST_) represents the energy difference between the lowest triplet (T_1_) and singlet (S_1_) excited states. Additionally, it is equal to the sum of ΔE_CT_ and ΔE_BET_ [30]. ΔE_CT_ means the drive force of charge-transfer (CT) state, and a small ΔE_CT_ can reduce the voltage loss to increase open-circuit voltage. ΔE_BET_ represents the difference between CT and T_1_ energies. By back electron transfer (BET), the triplet CT exciton can relax to the triplet state (T_1_) and charges can recombine. The reduced ΔE_BET_ can improve the speed of T_1_ to thermalize back into CT state and reduce the recombination of triplet charge transport excitons. As such, low ΔE_ST_ can effectively reduce the voltage loss in the exciton dissociation [31] and charge recombination through triplet state [20,30]. The results of ΔE_ST_ of the nine halogenated molecules calculated by Equations (16)–(18) were shown in Figure 8 and Table S3. The ΔE_ST_ generally increases with more halogen substitutions on account of the stronger electron-withdrawing ability of halogens (DRCN5T2F < DRCN5T4F < DRCN5T6F, DRCN5T2Cl < DRCN5T4Cl < DRCN5T6Cl, DRCN5T2Br < DRCN5T4Br < DRCN5T6Br). Additionally, the values of ΔE_ST_ for Cl- and Br-substituted molecules are higher than those of F-substituted molecules. The halogenated molecules DRCN5T2F, DRCN5T4F, DRCN5T6F, DRCN5T2Cl, and DRCN5T2Br have a similar level of ΔE_ST_ to DRCN5T (small ΔE_ST_ of 1.19 eV). Based on these results, they may be promising photovoltaic materials.

### 2.8. ESP of the Nine New Molecules

**Figure 9:**
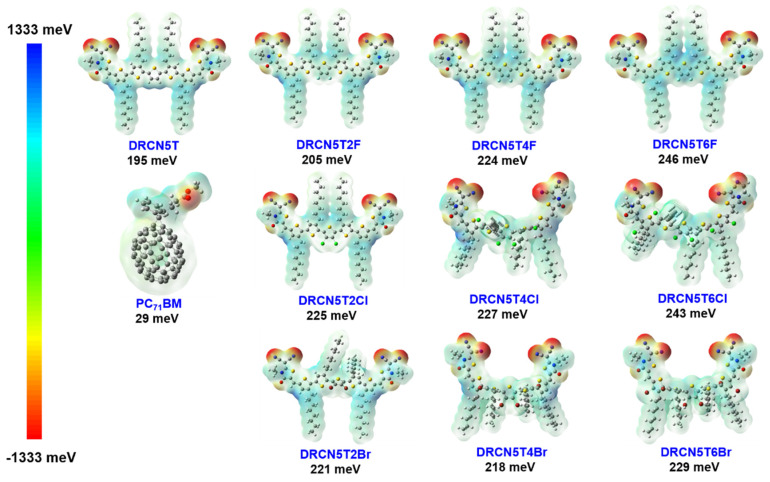
Averaged ESP of DRCN5T, DRCN5T2F, DRCN5T4F, DRCN5T6F, DRCN5T2Cl, DRCN5T4Cl, DRCN5T6Cl, DRCN5T2Br, DRCN5T4Br, DRCN5T6Br, and PC_71_BM calculated on B3LYP/6−31G(d) level.

It can be seen from Figure 9 that with more F and Cl substitutions, the electrostatic potential (ESP) rises. Additionally, as the ESP difference between the donor and the acceptor becomes bigger, the interaction between them increases [32]. The enhanced intermolecular interaction will increase the charge-transfer (CT) state ratio, but at the same time will cause a larger E_loss_, which is not conducive to photoelectric conversion efficiency [32]. Therefore, it is necessary to find a suitable number of F and Cl substitutions [33]. The tetrafluoro substitution is moderate with the averaged ESP of 224 meV. Dichloro and tetrachloro substitutions have almost the same averaged ESP, and their values are moderate. However, the ESP of Br-substituted molecules does not rise along with the increase in the number of substituted Br atoms. The averaged ESP (221 meV) of DRCN5T2Br is moderate. On the other hand, the averaged ESPs of DRCN5T2F, DRCN5T4F, DRCN5T6F, DRCN5T2Cl, DRCN5T4Cl, and DRCN5T2Br are higher than that of DRCN5T (195 meV), indicating that they are potential donors. Based on all the above results, the tetrafluoro substitution donor (DRCN5T4F) is the ideal donor among the difluoro, tetrafluoro, and hexafluoro substitution donors.

### 2.9. UV–Vis Spectra of the Designed New Molecules

**Figure 10:**
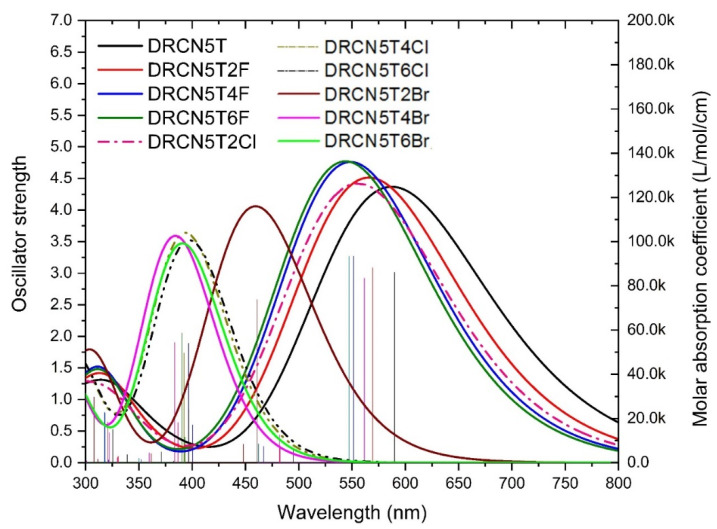
Calculated UV–Vis spectra of eight designed molecules obtained with CAM-B3LYP/6−31+ G(d) in PCM model.

**Figure 11:**
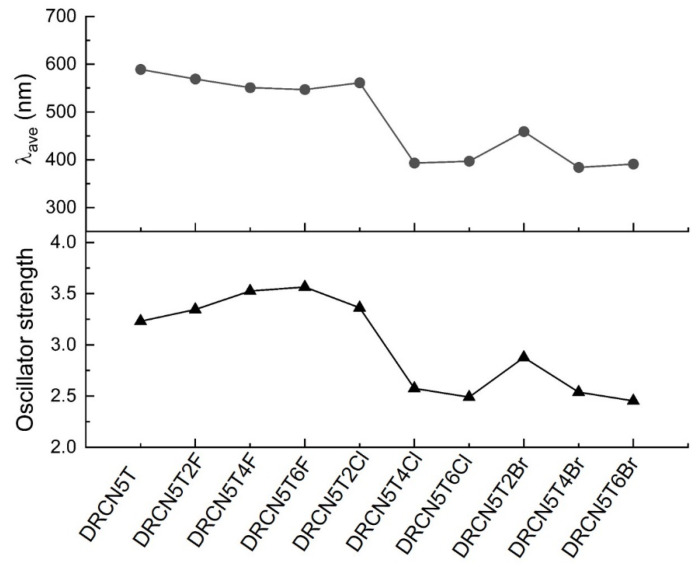
The averaged wavelength of absorption peaks and the total oscillator strength of the halogenated molecules.

As shown in Figure 10, UV–Vis spectra of DRCN5T4F, DRCN5T6F, DRCN5T2Cl, DRCN5T4Cl, DRCN5T6Cl, DRCN5T2Br, DRCN5T4Br, and DRCN5T6Br in chloroform with CAM-B3LYP functional are calculated. Table S4 shows the results of simulated excited states. For DRCN5T2F, DRCN5T4F, DRCN5T6F, DRCN5T2Cl, DRCN5T4Cl, DRCN5T6Cl, DRCN5T2Br, DRCN5T4Br and DRCN5T6Br, the maximum absorption peak is at 569 nm, 551 nm, 547 nm, 561 nm, 393 nm, 397 nm, 459 nm, 384 nm and 391 nm, respectively. The wavelength of these absorption peaks is blue-shifted compared to that of DRCN5T (Figure 10 and Figure 11) owing to the electron-withdrawing ability of halogens. As can be seen, the wavelength of DRCN5T4Cl, DRCN5T6Cl, DRCN5T2Br, DRCN5T4Br, and DRCN5T6Br blue-shifts a lot among these new molecules because of their larger fundamental energy gap (Figure 10 and Figure 11). Table S5 shows the calculated main absorption wavelength, main transition, and percentage of the transition. As can be seen from Table S5, for DRCN5T2F, DRCN5T4F, DRCN5T6F, and DRCN5T2Cl with planar structure, the main transition is HOMO to LMUO with percentage about 77%. Additionally, for non-planar DRCN5T4Cl, DRCN5T6Cl, DRCN5T2Br, DRCN5T4Br, and DRCN5T6Br, HOMO-1 to LUMO+1, HOMO to LUMO, HOMO-1 to LUMO and HOMO to LUMO+1 are the main transitions. 

The oscillator strength of DRCN5T4Cl, DRCN5T6Cl, DRCN5T4Br, and DRCN5T6Br decreases significantly compared with that of DRCN5T (Figure 11), which might be caused by their reduced planarity [25]. The oscillator strength of DRCN5T2F, DRCN5T4F, DRCN5T6F, and DRCN5T2Cl increases in contrast to that of DRCN5T. Based on these results, we may conclude that DRCN5T2F, DRCN5T4F, DRCN5T6F, and DRCN5T2Cl may be the better candidates for solar cells.

## 3. Methods

All molecular structures were built with GaussView version 5.0 [34]. Additionally, all calculations were calculated by using Guassian09 Rev E.01 software package [35]. To take into account environment effects, we used integral equation formalism variant polarizable continuum models (IEF-PCM) [36,37]. In order to compare with experiments, the energy of LUMO (lowest unoccupied molecular orbital), HOMO (highest occupied molecular orbital), and gap was computed in dichloromethane with a dielectric constant (ε) of 8.93, and the calculation of excited states for simulation of absorption spectra was carried out in chloroform with ε of 4.7113. As for thin film, ε was set to 3.0 since the values of dielectric constants of organic photovoltaic materials are around 3.0 [19,38]. DRCN5T, DRCN5TnF, DRCN5TnCl, and DRCN5TnBr (*n* = 2, 4, 6) molecules were optimized with the combination of B3LYP [38] hybrid density functional and 6−31G(d) basis set, which has been demonstrated as reliable for geometric optimization of organic molecules [39,40]. 6−31+G(d) basis set was selected for the rest of the calculations, and the added diffusion function is more accurate for weak interactions and excited states systems [41,42].

Firstly, we run benchmark calculations to obtain a suitable density functional by comparing absorption spectra with the available experimental data. CAM-B3LYP [43], ωB97X [44], and B3LYP density functionals were chosen. Among them, ωB97X and CAM-B3LYP are long range corrected (LRC) density functionals, which are more accurate in principle because they partly cure the derivative discontinuity problem and self-interaction error of traditional density functionals. 

In order to gain accurate HOMO/LUMO energy in solution, we calculated the energy of FMOs with the equations as follows [45]:

(1) $$\varepsilon_{H}^{\prime }=\varepsilon_{H}+\left[IP^{gas-phase}-IP^{PCM}\right]$$

(2) $$\varepsilon_{L}^{\prime }=\varepsilon_{L}+[EA^{gas-phase}-EA^{PCM}]\;$$

(3) $$IP^{gas-phase}=E\left(N-1\right)-E\left(N\right)\;$$

(4) $$EA^{gas-phase}=E\left(N\right)-E\left(N+1\right)\;$$

(5) $$IP^{PCM}=E^{PCM}\left(N-1\right)-E^{PCM}\left(N\right)$$

(6) $$EA^{PCM}=E^{PCM}\left(N\right)-E^{PCM}\left(N+1\right)$$

where $\varepsilon_{H\;}/\varepsilon_{L}$ represents the HOMO/LUMO energy of neutral system in gas phase at the equilibrium geometry obtained within PCM, respectively; $\varepsilon_{H}^{\prime }$/$\varepsilon_{L}^{\prime }$ represents the corrected HOMO/LUMO energy in condensed phase, respectively; N denotes the neutral system; EA/IP means electron affinity/ionization potential, respectively.

The system-dependent parameter ω of LRC density functional ωB97X [46] was obtained by using the following equations [46]:

(7) $$J^{2}\left(\omega \right)=J_{N}^{2}\left(\omega \right)+J_{N+1}^{2}\left(\omega \right)$$

(8) $$J_{N}^{2}\left(\omega \right)={\left[\varepsilon_{HOMO}^{\omega }\left(N\right)+E^{\omega }\left(N-1\right)-E^{\omega }\left(N\right)\right]}^{2}$$

(9) $$J_{N+1}^{2}\left(\omega \right)={\left[\varepsilon_{HOMO}^{\omega }\left(N+1\right)+E^{\omega }\left(N\right)-E^{\omega }\left(N+1\right)\right]}^{2}$$

where $E^{\omega }\left(N\right)$ is the energy of neutral system; $\varepsilon_{HOMO}^{\omega }\left(N\right)$ is the energy of HOMO of neutral system; $E^{\omega }\left(N+1\right)$ represents the energy of anion; $E^{\omega }\left(N-1\right)$ is the energy of cation. The optimized ωs are 0.08 Bohr^−1^ for both DRCN5T and DRCN5T2F. 

We roughly predicted open circuit voltages of OSCs with PC_71_BM as an acceptor by using the following empirical equation [26]:

(10) $$V_{OC}=\left(1/e\right)(|E_{HOMO}^{Donor}|-\left|E_{LUMO}^{PCBM}\right|)-0.3$$

where *e* is the electron charge; $E_{HOMO}^{Donor}$ means the energy of HOMO of donor; $E_{LUMO}^{PCBM}$ denotes the energy of LUMO of acceptor PC_71_BM. 

Multiwfn 3.8 software package was selected to simulate the absorption spectrum with the output of 40 excited states [47]. Additionally, the full width at half maximum (FWHM) of absorption peak was default (0.6667 eV). For the wavelength of simulated absorption peaks, the following equation was used:

(11) $$\lambda_{ave}=\frac{\sum^{\;}\lambda_{n}\times f_{n}}{\sum^{\;}f_{n}}$$

where $\lambda_{ave}$ represents the wavelength of absorption peak; $f_{n}$ denotes the oscillator strength of the nth excited state; $\lambda_{n}$ is the wavelength of the nth excited state.

The following equation was used to calculate the inner reorganization energy [28,48]:

(12) $$\;\lambda_{i,\;h}=\left[E^{0}\left(D^{+}\right)-E^{0}\left(D^{0}\right)\right]+{\left[E^{+}\left(D^{0}\right)-E^{+}\left(D^{+}\right)\right]}^{\;}$$

where $\;\lambda_{i,\;h}$ is the inner reorganization energy; $E^{0}\left(D^{+}\right)$ is the total energy of neutral donor in the optimized geometry of cation; $E^{0}\left(D^{0}\right)$ represents the total energy of neutral donor in the optimized geometry of neutral molecule; $E^{+}\left(D^{0}\right)$ denotes energy of positive donor in the optimized geometry of neutral molecule; $E^{+}\left(D^{+}\right)$ is energy of positive donor in the optimized geometry of cation. 

Exciton binding energy is a key factor in influencing charge transfer and separation. The following equations were used to calculate exciton binding energy with IEF-PCM [49,50]:

(13) $$E_{b}=E_{g}^{fund}-E_{g}^{opt}\;$$

(14) $$E_{g}^{fund}=E\left(N-1\right)+E\left(N+1\right)-2E\left(N\right)\;$$

(15) $$E_{g}^{opt}=E\left(N,Excited\right)-E\left(N\right)\;\;$$

where $E_{b}$ is exciton binding energy; $E_{g}^{opt}$ denotes the energy of optical gap; $E_{g}^{fund}$ is the energy of fundamental gap; *N*/*N* + 1/*N* − 1 represents a neutral/anion/cation system, respectively.

The singlet–triplet energy gap ($\Delta E_{ST}$) is an important parameter for organic photovoltaic materials and denotes the energy difference between the lowest singlet (S_1_) and triplet (T_1_) excited states [30]. $\Delta E_{ST}$ was calculated as follows [51].

(16) $$\Delta E_{ST}=E_{S1}-E_{T1}=\Delta E_{CT}+\Delta E_{BET}$$

(17) $$\Delta E_{CT}=E_{S1}-E_{CT}$$

(18) $$\Delta E_{BET}=E_{CT}-E_{T1}$$

## 4. Conclusions

By using DFT and TDDFT calculations, we have studied the effects of halogen substitution on the physical and opto-electronic properties of these new molecules. From DRCN5T2F to DRCN5T4F to DRCN5T6F, the dipole moment first increases and then decreases. For Cl- and Br-substituted molecules, their dipole moments have the same tendency as F-substituted molecules. Halogenated molecules with a slightly smaller dipole moment than that of DRCN5T possibly enhance fill factor and PCE of OSCs. The HOMO energy of DRCN5T4Cl, DRCN5T6Cl, DRCN5T2Br, DRCN5T4Br, and DRCN5T6Br greatly reduces due to their non-planarity, which leads to a significant increase in their V_OCS_. The lowest inner reorganization energy of DRCN5T6F may indicate the smallest energy loss. The exciton binding energy (E_b_) of fluorine-substituted molecules rises with more fluorine substitutions. For Cl- and Br-substituted molecules, with the increase in the number of substitutions, E_b_ increases at first and then reduces. DRCN5T2Cl exhibits the smallest value of E_b_, indicating that it may be a potential donor material. DRCN5T2F, DRCN5T4F, DRCN5T6F, DRCN5T2Cl, and DRCN5T2Br have a similar ΔE_ST_ to DRCN5T, demonstrating that they may be promising photovoltaic materials. DRCN5T4F may be the ideal donor among the difluoro, tetrafluoro, and hexafluoro substitutions donors based on the moderate averaged ESP. Compared to DRCN5T, DRCN5T2F, DRCN5T4F, DRCN5T6F, and DRCN5T2Cl possess the enhanced oscillator strength of UV–Vis spectra. In summary, we may conclude that DRCN5T2Cl, DRCN5T4F, and DRCN5T6F have the potential to be good donors. Our work may provide a guideline for future halogenation modification of DRCN5T and lay the foundations for the development of oligothiophene-based donors.

## Figures and Tables

**Table 1:** The experimental and calculated absorption peaks in the visible region and corresponding energy of DRCN5T and DRCN5T2F.

DRCN5T		Experiment	CAM-B3LYP	ωB97X	B3LYP
	λ_max_ (nm)	531	589	688	766
	energy (eV)	2.34	2.11	1.80	1.62
DRCN5T2F		Experiment	CAM-B3LYP	ωB97X	B3LYP
	λ_max_ (nm)	500	569	663	731
	energy (eV)	2.48	2.18	1.87	1.70

**Table 2:** The predicted V_OC_ of OSCs with DRCN5T and its derivatives as donor and PC_71_BM as acceptor via Equation (10). EA/IP correction denotes the absolute value of EA_D, film_-EA_D, vacuum_ or IP_D, film_-IP_D, vacuum_, respectively. Unit: V for V_OC_; eV for energy. Cor: Corrected; Exp: Experimental. In order to be consistent with the experimental value, we make corrections to all calculated V_OC_s, i.e., Cor V_OC_ = V_OC_ − 1.33 V, in which 1.33 V is the V_OC_ deviation between experiment and calculation of DRCN5T/PC_71_BM OSC.

	DRCN-5T	DRCN-5T2F	DRCN-5T4F	DRCN-5T6F	DRCN-5T2Cl	DRCN-5T4Cl	DRCN-5T6Cl	DRCN-5T2Br	DRCN-5T4Br	DRCN-5T6Br
HOMO	−6.43	−6.57	−6.74	−6.90	−7.22	−7.65	−7.78	−7.04	−7.73	−7.81
LUMO	−2.49	−2.53	−2.57	−2.69	−2.34	−2.24	−2.36	−2.25	−2.21	−2.34
IP correction	0.80	0.79	0.80	0.83	0.79	0.81	0.85	0.79	0.79	0.87
EA correction	0.50	0.49	0.47	0.44	0.72	0.50	0.49	0.72	0.52	0.51
V_OC_	2.32	2.47	2.63	2.76	3.12	3.53	3.62	2.94	3.62	3.63
Cor V_OC_	0.99	1.14	1.30	1.43	1.79	2.20	2.29	1.61	2.29	2.30
Exp V_OC_	0.99 [7]	1.12 [17]	N/A	N/A	N/A	N/A	N/A	N/A	N/A	N/A

## Data Availability

Not applicable.

## Supplementary Materials

The following are available online at https://www.mdpi.com/article/10.3390/ijms222413498/s1.
