# Supplementary material for: Designing Potential Donor Materials Based on DRCN5T with Halogen Substitutions: A DFT/TDDFT Study
Source: Int J Mol Sci. 2021 Dec 16;22(24):13498. doi: 10.3390/ijms222413498 (PMC8704226; doi:10.3390/ijms222413498)
Supplement: Supplementary file 1 [file ijms-22-13498-s001.zip › ijms-1451672-supplementary.pdf]

Supporting Information:

Designing potential donor materials based on DRCN5T with halogen substitutions: A DFT/TDDFT study

Yunjie Xiang*, Jie Zhang and Shaohui Zheng*

School of Materials and Energy

Chongqing Key Laboratory for Advanced Materials and Technologies of Clean Energies

Southwest University, Chongqing, China

Corresponding author: hao1986kx88@swu.edu.cn; shaohuizheng@swu.edu.cn

Table S1. Calculated excited states and oscillator strength of DRCN5T and DRCN5T2F in chloroform by using different functionals.

		Excited State	Energy (eV)	λ(nm)	Oscillator strength
DRCN5T	B3LYP	1	1.6136	768.39	2.5626
		2	1.9168	646.81	0.0412
		λ_{ave}	1.6178	766.47	2.6038
		8	3.1708	391.02	0.1890
		9	3.3361	371.65	0.0066
		10	3.3764	367.20	0.0047
		11	3.472	357.09	0.3960
		14	3.5674	347.55	0.0118
		15	3.6014	344.26	0.0172
		16	3.6182	342.67	0.0020
		17	3.6423	340.40	0.0054
		19	3.6943	335.61	0.1793
		20	3.7227	333.05	0.0034
		21	3.8202	324.55	0.0001
		22	3.8208	324.50	0.0660
		λ_{ave}	3.4723	357.11	0.8815

	CAM-B3LYP	1	2.1035	589.41	3.0149
		λ_{ave}	2.1035	589.41	3.0149
		5	3.6601	338.75	0.1300
		6	3.8116	325.28	0.5263
		7	4.0947	302.79	0.0041
		8	4.1511	298.68	0.3616
		9	4.2492	291.78	0.0019
		10	4.2609	290.98	0.0166
		λ_{ave}	3.9114	317.02	1.0405
	ωB97X	1	1.8030	687.66	2.9121
		λ_{ave}	1.8030	687.66	2.9121
		6	3.4047	364.16	0.0003
		7	3.4393	360.49	0.0135
		8	3.5025	353.99	0.6091
		9	3.6839	336.55	0.0062
		10	3.7069	334.47	0.0357
		11	3.7213	333.17	0.0044
		14	3.7753	328.41	0.0956
		15	3.7932	326.86	0.0131
		16	3.8418	322.73	0.0114
		λ_{ave}	3.5533	348.97	0.7893
		Excited State	Energy (eV)	λ(nm)	Oscillator strength
DRCN5T2F	B3LYP	1	1.6901	733.60	2.6352
		2	2.0034	618.88	0.0558
		λ_{ave}	1.6958	731.22	2.6910
		8	3.2266	384.25	0.1249
		9	3.3436	370.81	0.001
		10	3.4377	360.66	0.0092
		11	3.4792	356.36	0.4150
		12	3.5894	345.42	0.0101
		15	3.6137	343.09	0.0566

	16	3.6348	341.11	0.0007
	17	3.6499	339.69	0.0021
	18	3.6674	338.07	0.0793
	19	3.7244	332.90	0.0075
	20	3.7322	332.20	0.0465
	21	3.7993	326.34	0.0156
	λ_{ave}	3.4871	355.60	0.7685
CAM-B3LYP	1	2.1797	568.81	3.0861
	λ_{ave}	2.1797	568.81	3.0861
	5	3.7523	330.42	0.1005
	6	3.8681	320.53	0.6448
	7	4.1244	300.61	0.0103
	8	4.1785	296.72	0.2691
	9	4.251	291.66	0.0008
	λ_{ave}	3.9361	315.03	1.0255
ωB97X	1	1.8693	663.27	2.9818
	λ_{ave}	1.8693	663.27	2.9818
	6	3.4634	357.99	0.0342
	7	3.4638	357.95	0.0846
	8	3.5336	350.87	0.5141
	9	3.703	334.82	0.0087
	10	3.7083	334.34	0.0484
	11	3.7265	332.71	0.0004
	12	3.7883	327.28	0.1387
	13	3.7913	327.03	0.0003
	15	3.8255	324.1	0.0101
	16	3.8645	320.83	0.0192
	λ_{ave}	3.5844	345.94	0.8587

	DRCN5T2F		DRCN5T4F		DRCN5T6F	
**Vertical**	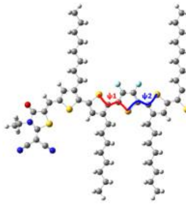		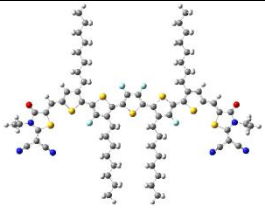		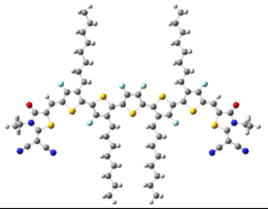	
**Side**	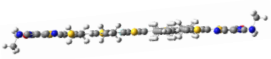		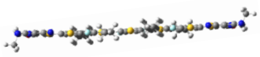		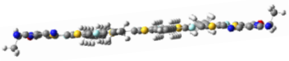	
**Dihedral angle**	ψ1=0°	Ψ2=0°	ψ1=0°	Ψ2=2°	ψ1=1°	Ψ2=0°
	**DRCN5T2Cl**		**DRCN5T4Cl**		**DRCN5T6Cl**	
**Vertical**	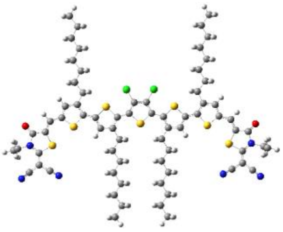		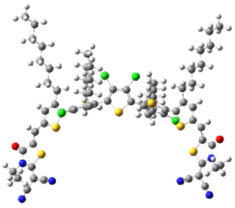		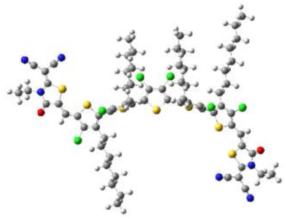	
**Side**	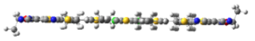		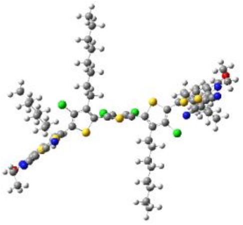		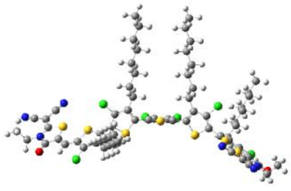	
**Dihedral angle**	ψ1=0°	Ψ2=1°	ψ1=80°	Ψ2=-80°	ψ1=77°	Ψ2=-78°
	**DRCN5T2Br**		**DRCN5T4Br**		**DRCN5T6Br**	
**Vertical**	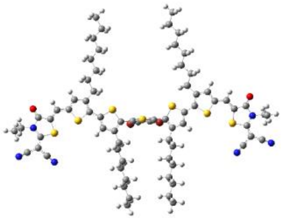		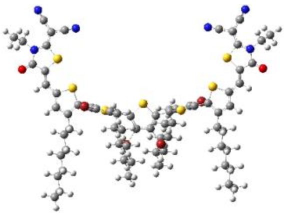		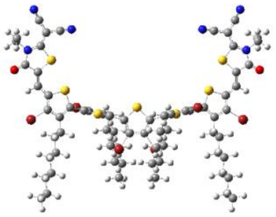	
**Side**	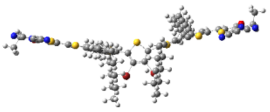		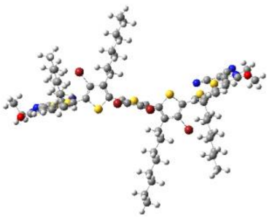		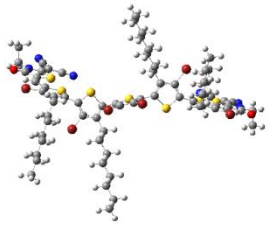	
**Dihedral angle**	ψ1=86°	Ψ2=86°	ψ1=89°	Ψ2=89°	ψ1=90°	Ψ2=89°

Figure S1. The molecular structures in vertical and side view and illustration of selected dihedral angle of DRCN5T2F, DRCN5T4F, DRCN5T6F, DRCN5T2Cl, DRCN5T4Cl, DRCN5T6Cl, DRCN5T2Br, DRCN5T4Br and DRCN5T6Br.

Table S2. Exciton binding energy of DRCN5T, DRCN5T2F, DRCN5T4F, DRCN5T6F, DRCN5T2Cl, DRCN5T4Cl, DRCN5T6Cl, DRCN5T2Br, DRCN5T4Br and DRCN5T6Br with CAM-B3LYP/6-31+G(d) in film (ε = 3.0). IP/EA mean ionization potential/electron affinity respectively; E_{opt} denotes the energy of optical gap.

	DRCN5T	DRCN5T2F	DRCN5T4F	DRCN5T6F	DRCN5T2Cl
IP (eV)	5.51	5.67	5.85	5.99	6.06
EA (eV)	2.87	2.90	2.94	3.04	2.89
Fundamental gap (IP – EA) (eV)	2.64	2.77	2.91	2.95	3.17
E_{opt} (eV)	2.12	2.19	2.27	2.28	2.72
Exciton binding energy (eV)	0.52	0.58	0.64	0.67	0.45
	DRCN5T4Cl	DRCN5T6Cl	DRCN5T2Br	DRCN5T4Br	DRCN5T6Br
IP	6.55	6.66	6.19	7.00	6.80
EA	2.54	2.66	2.93	2.55	2.69
Fundamental gap (IP – EA) (eV)	4.01	4.00	3.26	4.45	4.11
E_{opt} (eV)	3.14	3.14	2.70	3.22	3.17
Exciton binding energy (eV)	0.87	0.86	0.56	1.23	0.94

Table S3. The lowest singlet (E_{S1}), triplet excited energy (E_{T1}) and singlet-triplet energy gap (ΔE_{ST}) of DRCN5T, DRCN5T2F, DRCN5T4F, DRCN5T6F, DRCN5T2Cl, DRCN5T4Cl, DRCN5T6Cl, DRCN5T2Br, DRCN5T4Br and DRCN5T6Br. Unit: eV for E_{S1}, E_{T1} and ΔE_{ST}.

System	E_{S1}	E_{T1}	ΔE_{ST}
DRCN5T	2.12	0.93	1.19
DRCN5T2F	2.19	0.99	1.20
DRCN5T4F	2.26	1.05	1.22
DRCN5T6F	2.28	1.06	1.23
DRCN5T2Cl	2.72	1.39	1.33
DRCN5T4Cl	3.14	1.69	1.44
DRCN5T6Cl	3.14	1.65	1.49
DRCN5T2Br	2.70	1.36	1.34
DRCN5T4Br	3.22	1.71	1.51
DRCN5T6Br	3.17	1.67	1.50

Table S4. Calculated excited states and oscillator strength of DRCN5T, DRCN5T2F, DRCN5T4F, DRCN5T6F, DRCN5T2Cl, DRCN5T4Cl, DRCN5T6Cl, DRCN5T2Br, DRCN5T4Br and DRCN5T6Br in chloroform by using CAM-B3LYP functional.

	Excited State	Energy (eV)	λ(nm)	Oscillator strength
DRCN5T	1	2.1035	589.41	3.0149
	λ_{ave}	2.1035	589.41	3.0149
	5	3.6601	338.75	0.1300
	6	3.8116	325.28	0.5263
	7	4.0947	302.79	0.0041
	8	4.1511	298.68	0.3616
	9	4.2492	291.78	0.0019
	10	4.2609	290.98	0.0166
	λ_{ave}	3.9114	317.02	1.0405
DRCN5T2F	1	2.1797	568.81	3.0861
	λ_{ave}	2.1797	568.81	3.0861
	5	3.7523	330.42	0.1005
	6	3.8681	320.53	0.6448
	7	4.1244	300.61	0.0103
	8	4.1785	296.72	0.2691
	9	4.251	291.66	0.0008
	λ_{ave}	3.9361	315.03	1.0255
DRCN5T4F	1	2.2503	550.96	3.2693
	λ_{ave}	2.2503	550.96	3.2693
	5	3.8601	321.2	0.0437
	6	3.8998	317.93	0.7914
	7	4.1666	297.57	0.0131
	8	4.1969	295.42	0.2437
	9	4.3028	288.15	0.0164
	λ_{ave}	3.9689	312.43	1.1083
DRCN5T6F	1	2.2681	546.65	3.2666
	λ_{ave}	2.2681	546.65	3.2666

	5	3.8906	318.67	0.0184
	6	3.9055	317.46	0.7964
	7	4.18	296.61	0.1674
	8	4.1905	295.87	0.02
	9	4.26	291.04	0.0192
	λ_{ave}	3.9598	313.15	1.0214
DRCN5T2Cl	1	2.2096	561.11	2.9216
	λ_{ave}	2.2096	561.11	2.9216
	6	3.8493	322.09	0.4723
	7	4.1217	300.81	0.0303
	8	4.173	297.11	0.2786
	9	4.2517	291.61	0.0007
	10	4.2925	288.84	0.0071
	11	4.348	285.15	0.0018
	13	4.3681	283.84	0.0002
	14	4.4159	280.77	0.2629
	15	4.4287	279.96	0.0164
	λ_{ave}	4.0806	303.88	1.0703
DRCN5T4Cl	1	3.1272	396.47	0.8408
	2	3.1633	391.95	1.7348
	λ_{ave}	3.1518	393.43	2.5756
	3	4.1882	296.03	0.5324
	4	4.206	294.78	0.3742
	5	4.3272	286.52	0.0577
	6	4.3345	286.04	0.0031
	7	4.521	274.24	0.0249
	8	4.5282	273.8	0.0471
	9	4.533	273.51	0.0631
	10	4.5347	273.41	0.1536
	11	4.6677	265.62	0.201
	12	4.6747	265.22	0.0029
	λ_{ave}	4.3262	286.62	1.4600

DRCN5T6Cl	1	3.0982	400.18	0.6003
	2	3.1297	396.15	1.8899
	λ_{ave}	3.1225	397.12	2.4902
	3	4.1463	299.02	0.4888
	4	4.1592	298.09	0.1977
	5	4.2317	292.99	0.132
	6	4.2384	292.53	0.0706
	7	4.4637	277.76	0.1256
	8	4.4646	277.71	0.1294
	9	4.5346	273.42	0.0733
	11	4.619	268.42	0.0213
	12	4.6192	268.41	0.0038
	13	4.6585	266.15	0.2012
	λ_{ave}	4.3078	287.85	1.4437
DRCN5T2Br	1	2.6928	460.43	2.5857
	2	2.7701	447.58	0.2922
	λ_{ave}	2.7008	459.13	2.8779
	3	3.9823	311.34	0.0563
	4	4.0291	307.72	1.0364
	5	4.2192	293.85	0.0062
	6	4.2265	293.35	0.02
	7	4.249	291.79	0.0733
	8	4.2662	290.62	0.0124
	9	4.3713	283.63	0.0038
	10	4.403	281.59	0.0046
	11	4.406	281.4	0.0004
	λ_{ave}	4.0488	306.26	1.2134
DRCN5T4Br	1	3.2090	386.36	0.6348
	2	3.2351	383.25	1.9037
	λ_{ave}	3.2289	384.03	2.5385
	13	4.7795	259.41	0.096
	14	4.7894	258.87	0.0377

	16	4.8552	255.36	0.0084
	17	4.9723	249.35	0.0778
	18	4.977	249.12	0.0307
	19	5.0013	247.9	0.0709
	20	5.0096	247.5	0.0097
	21	5.0239	246.79	0.0958
	22	5.024	246.78	0.2275
	23	5.0678	244.65	0.4315
	24	5.0751	244.3	0.0005
	25	5.138	241.31	0.1618
	26	5.1452	240.97	0.1577
	27	5.1951	238.66	0.0031
	28	5.2291	237.11	0.007
	29	5.2551	235.93	0.0201
	30	5.2611	235.66	0.2432
	31	5.2879	234.47	0.0062
	32	5.3427	232.06	0.0021
	33	5.3507	231.71	0.0004
	λ_{ave}	5.0704	244.56	1.6881
DRCN5T6Br	1	3.1556	392.90	0.4029
	2	3.1786	390.06	2.0507
	λ_{ave}	3.1752	390.53	2.4536
	13	4.7277	262.25	0.0274
	14	4.7332	261.94	0.0169
	15	4.7657	260.16	0.0003
	16	4.7727	259.78	0.0034
	17	4.8973	253.17	0.0155
	18	4.8989	253.08	0.0234
	19	4.937	251.13	0.059
	20	4.945	250.73	0.001
	21	5.0203	246.97	0.9761
	22	5.0686	244.61	0.0023

24	5.1004	243.09	0.0312
25	5.1244	241.95	0.1863
26	5.1337	241.51	0.1505
28	5.1768	239.5	0.0035
29	5.1839	239.17	0.0031
30	5.2289	237.11	0.0042
31	5.2335	236.91	0.2513
32	5.2707	235.23	0.0018
33	5.2732	235.12	0.0233
34	5.3298	232.62	0.0025
35	5.361	231.27	0.0001
λ_{ave}	5.0626	244.93	1.7831

Table S5. Calculated wavelength of the main absorption, main transition and contribution to the transition of DRCN5T, DRCN5T2F, DRCN5T4F, DRCN5T6F, DRCN5T2Cl, DRCN5T4Cl, DRCN5T6Cl, DRCN5T2Br, DRCN5T4Br and DRCN5T6Br in chloroform by using CAM-B3LYP functional.

Molecule	Wavelength (nm)	Type of transition	Percentage
DRCN5T	589	HOMO—LUMO	77%
DRCN5T2F	569	HOMO—LUMO	76%
DRCN5T4F	551	HOMO—LUMO	77%
DRCN5T6F	547	HOMO—LUMO	77%
DRCN5T2Cl	561	HOMO—LUMO	75%
DRCN5T4Cl	393	HOMO-1—LUMO+1	46%
		HOMO—LUMO	47%
		HOMO-1—LUMO	47%
		HOMO—LUMO+1	47%
DRCN5T6Cl	397	HOMO-1—LUMO+1	46%
		HOMO—LUMO	48%
		HOMO-1—LUMO	47%
		HOMO—LUMO+1	47%
DRCN5T2Br	459	HOMO-1—LUMO+1	37%
		HOMO—LUMO	54%
		HOMO-1—LUMO	39%
		HOMO—LUMO+1	44%
DRCN5T4Br	384	HOMO-1—LUMO+1	47%

		HOMO—LUMO	48%
		HOMO-1—LUMO	48%
		HOMO—LUMO+1	48%
DRCN5T6Br	391	HOMO-1—LUMO+1	47%
		HOMO—LUMO	48%
		HOMO-1—LUMO	48%
		HOMO—LUMO+1	48%
